# Analysis of Ultrasonographic Characteristics of Early Hepatic Alveolar Echinococcosis

**DOI:** 10.3389/fsurg.2022.918138

**Published:** 2022-07-05

**Authors:** Yong-Xing Wang, Wei Liu, Zhan-Yong Sun, Lan Wu, Xian-Kun Xie, Bo Liu

**Affiliations:** ^1^ Department of Surgery, The 969th Hospital of the PLA Joint Logistics Support Force, Hohhot, China; ^2^ Department of Ultrasound, The 969th Hospital of the PLA Joint Logistics Support Force, Hohhot, China; ^3^ Department of Ultrasound, Jiuzhi People’s Hospital, Zhiqingsongduo, China

**Keywords:** alveolar echinococcosis, liver, ultrasonogram, characteristics, concomitant sign

## Abstract

**Objective:**

This study aims to investigate the ultrasonographic characteristics of early hepatic alveolar echinococcosis (HAE) and improve the qualitative diagnostic ability of sonographers.

**Methods:**

The data of 80 positive cases of HAE screened and diagnosed by ultrasonography and serum immunology (33 males and 44 females with a mean age of 44.12 ± 14.31 y) were used in the study. The imaging characteristics of the lesions (i.e., intrahepatic distribution, shape, size, echo, blood flow, and growth characteristics) were analyzed retrospectively, and the ultrasonographic characteristics of early lesions were discussed in combination with their basic pathological changes.

**Results:**

Patients with lesions of the proliferative infiltration type accounted for 57.5% (46/80), the fibrous calcification type accounted for 26.25% (21/80), the necrotic liquefaction type accounted for 6.25% (5/80), and the mixed type accounted for 10% (8/80). Patients with lesions involving the right lobe and the left lobe accounted for 76.25% (61/80) and 11.25% (9/80), respectively, and with lesions involving both the left and right lobes accounted for 12.5% (10/80). There were statistically significant differences in diameter between all types of lesions (*n* = 88; F = 5.926 and *P *= 0.004). Focal hyperechoic and diffuse heterogenous nodular changes were the main manifestations of early proliferative infiltration lesions.

**Conclusion:**

Ultrasonography is extremely valuable in the diagnosis of early HAE. Capillary hemangioma-like changes and hailstorm sign are the main characteristics of early lesions of HAE, and calcification is a common concomitant sign.

## Introduction

Echinococcosis (also kn own as hydatid disease) is a zoonotic parasitic disease caused by the metacestode of *Echinococcus spp*. The prevention and control of echinococcosis has been recognized as a major global public health problem (1). Some research pointed out that echinococcosis caused damage to patients’ health and increased financial burden in both developed and developing countries such as Iraq, India and America (2–4). As for China, there are two main types of echinococcosis prevalent: cystic echinococcosis, also known as echinococcosis granulosa, which is common in epidemic areas, and sporadic cases have been reported on the mainland, and alveolar echinococcosis (AE), also known as echinococcosis multilocularis, which is rare. The primary lesion of AE often occurs in the liver (5). This is called hepatic alveolar echinococcosis (HAE) and is primarily prevalent in the pure pastoral areas of the Qinghai-Tibet Plateau but is rare on the mainland. The five provinces in Western China are the main endemic areas of echinococcosis (6), of which several counties in the south of Qinghai Province are the hardest hit areas (7, 8).

Jiuzhi county is in the Sanjiangyuan area in the south of Qinghai Province. Due to the unique geographical environment, climatic conditions, religious customs, poor sanitary conditions, and poor living habits, this area has become a high-risk area for echinococcosis in China, especially AE. The 2012 national echinococcosis prevalence survey showed that the echinococcosis prevalence in the population in this area was as high as 12.83% (9). Foxes are considered to be the final host supporting the life cycle of *Echinococcus multilocularis* Leuckart in the environment, dogs are a possible final host, and humans are only occasionally intermediate hosts. *Echinococcus* eggs that are accidentally eaten are incubated into oncospheres in the duodenum, pass through the small veins of the intestinal mucosa and enter the portal vein, form primary lesions in the liver, inflitrate the surrounding normal tissues by exogenous budding, and grow slowly. The asymptomatic incubation period of AE in patients is approximately 5–15 y, and the eggs can travel through the body via blood and lymph or invade adjacent organs directly (10–12). When the lesions increase in size or produce metastatic symptoms, most patients are in the intermediate or advanced stages. Studies have shown that in the absence of treatment, the fatality rate for patients with AE within 10 y is >95%, and AE has been recognized as the most fatal zoonotic parasitic disease (13, 14).

As a convenient, fast, and efficient imaging examination, ultrasonography is considered to be the first choice for the diagnosis of echinococcosis (15, 16), especially in large-scale population screening (14, 15, 17, 18). Previous imaging studies primarily used inpatient or outpatient cases as samples, with the majority of patients in the middle and late stages of the disease, and the surgical resection rate was approximately 30% (19). Therefore, early diagnosis has important practical significance for patients with HAE (20). Mecit Kantarci tried to find a feasible way to diagnose patients with HEA earlier (21) but the number of patients in the study is relatively small and the research on HEA look forward to cover more patients. To date, few large-sample imaging studies have been conducted on the early characteristics of HAE. In addition, WHO-IWGE has not proposed a classification standard, either using ultrasonography or CT, that is as widely accepted as the CE classification. Therefore, we used domestic standards in this study. In all types of cases, the lesions mainly involved the right lobe of the liver, which is consistent with previous research results. The reason for this may be related to the fact that *Echinococci* invade the liver through the portal vein, and the right branch of the liver supplied by the portal vein is dominant. In a study on 185 outpatients with HAE, Kratzer et al.(22) considered HAE lesions with hailstorm sign as early characteristic manifestations, with typical ultrasonographic manifestations of “hyperechoic nodules with poorly defined boundary, irregular shape and uneven internal echo, with/without acoustic shadow.” In this study, the lesions with the above signs were found in both the AE1 and AE2 types, but we found that compared with focal hyperechoic nodules with smaller diameter, they should be an earlier manifestation of HAE, which is called “capillary hemangioma”-like change. The shape of the lesions was not smooth, and they may have exhibited angulation deformity, rough internal echo spots, and punctate strong echo or been followed by a faint acoustic shadow. These characteristics are distinguishable from intrahepatic hemangioma, regenerative nodules, and inflammatory nodules.

In September 2017, our Tibet-supporting medical team went to Jiuzhi County, Golog Prefecture, Qinghai Province to conduct the screening task for echinococcosis that was coordinated by the state. During this period, we collected and sorted 80 cases of HAE with complete data. In this paper, we analyze and summarize their ultrasonographic characteristics and report as follows.

## Materials and Methods

### Demographic Data

All cases in this paper are patients who were screened for echinococcosis in Baiyu Township, Waeryi Township, and Zhiqingsongduo Town, Jiuzhi County. Except for one Han who had lived for a long period in Tibetan areas, all patients were local Tibetans, including herdsmen (81.25%, 65/80), religious people (15%, 12/80), and township residents (3.75%, 3/80). The male to female (M/F) ratio was 1:1.22, with males accounting for 45% (36/80) and females for 55% (44/80), with a mean age of 44.12 ± 14.31 y (7–75 y). All cases included were positive for HAE based on screening via both ultrasonography and immunology.

### Methods

The instruments used were the Mindray portable color ultrasound machine S5 and GE Logiq S7 (both equipped with an abdominal probe with a frequency of 3.5–5.5 MHz). The patients fasted for more than eight hours before the examination. A transabdominal multi-section scan was performed on each patient’s liver while the patient was in the supine position or the left or right lateral position. The size, number, intrahepatic distribution, echo characteristics, and blood flow information of the lesions were measured and recorded. The proximal superficial lesions were observed using a high-frequency probe, and the static or dynamic images were saved. Finally, a routine full abdominal scan was performed. For immunological testing, we used the echinococcus antibody enzyme-linked immunosorbent assay test kit (Shenzhen Combined Biotech Co., Ltd.) according to the instructions on the kit to detect anti-echinococcus immunoglobulin G antibody.

With regard to case diagnosis, we referred to the diagnostic criteria for AE cases determined by the World Health Organization Informal Working Groups on Echinococcosis (WHO-IWGE): (1) life history in the epidemic area and typical imaging changes, (2) presence of evidence on immunological laboratory examination, and (3) observed evidence of echinococcosis development or the presence of a basis for pathological diagnosis (8). Clinical diagnosis can be made if two of the above criteria are met. All 80 cases in this paper met the first two of these criteria.

Ultrasonographic classification was carried out according to the Chinese standard *Diagnostic Criteria for Echinococcosis* (WS257-2006). The ultrasonograms were analyzed by two senior attending physicians majoring in ultrasonographic imaging, and their consensus was taken as the final classification result. In addition to the 80 AE cases in this paper, 3 cases were abandoned due to a lack of unified diagnostic opinion.

### Statistical Analysis

Enumeration data are expressed as percentages (%), measurement data are expressed in mean ± standard deviation $\left(\bar{x}\pm s\right)$, and all data were processed using statistical software (SPSS 20.0). The Bonferroni method in one-way analysis of variance was used for comparison between groups, and *P *< 0.05 was considered statistically significant.

## Results

### Statistical Analysis of Cases

Of the 80 HAE cases included in this study, patients with lesions of the proliferative infiltration type (AE1) accounted for 57.5% (46/80), with an M/F ratio of 19:27 and age 45.73 ± 14.61 y; the fibrous calcification type (AE2) accounted for 26.25% (21/80), with an M/F ratio of 8:13 and age 41.14 ± 14.32 y; the necrotic liquefaction type (AE3) accounted for 6.25% (5/80), with an M/F ratio of 4:1 and age 41.00 ± 6.67 y; and mixed types accounted for 10% (8/80), with an M/F ratio of 5:3 and age 45.63 ± 16.76 y, including six cases with AE1 + AE2 mixed lesions and two cases with AE1 + AE3 mixed lesions. In all types, cases with lesions involving only the right or left lobe accounted for 76.25% (61/80) and 11.25% (9/80), respectively, and cases with lesions involving both the left and right lobes accounted for 12.5% (10/80). The right lobe of the liver was most involved in all types but was more obvious in AE1 and AE2 types (see Table 1).

**Table 1:** Ultrasonographic classification and involved hepatic lobe of 80 HAE cases (n/cases).

Hepatic lobe	Ultrasonographic classification				Total
	AE_1_	AE_2_	AE_3_	Mixed type	
R	32	18	5	6	61
L	7	2	0	0	9
R + L	7	1	0	2	10
Total	46	21	5	8	80

*AE_1_*, *proliferative infiltration type; AE_2_*, *fibrous calcification type; AE_3_*, *necrotic liquefaction type; R*, *right lobe of liver; L*, *left lobe of liver.*

The maximum diameter of the 88 lesions was statistically analyzed (for the same cases, the lesion with the maximum diameter of each type was included in the study). The diameter of the lesions was 51.73 ± 33.99 mm (range: 9–170 mm). Significant differences in diameter existed between the three types: AE1 (54/88, 55.48 ± 35.95 mm), AE2 (27/88, 36.89 ± 20.12 mm), AE3 (7/88, 80.00 ± 39.16 mm; F = 5.926 and *P *= 0.004), AE2 + AE1 (*P *= 0.049), and AE2 + AE3 (*P *= 0.007; see Table 2).

**Table 2:** Maximum diameter and ultrasonographic classification of 88 HAE lesions (n/lesions).

Maximum diameter (mm)	AE_1_			AE_2_	AE_3_
	AE_1_a	AE_1_b	AE_1_c		
<50	16	9	5	21	1
50–100	0	8	10	6	5
>100	0	2	4	0	1

*AE_1_a, focal type; AE_1_b, diffuse type; AE_1_c, mass type.*

### Ultrasonographic Manifestation

The proliferative infiltration type had various manifestations and different echo levels. According to shape, size, and growth characteristics, this type can be further divided into three categories: (1) The focal type (18.18%, 16/88) has a quasi-circular or irregular shape, poorly defined boundary, small size (<5 cm diameter), and capillary hemangioma-like changes. The hyperechoic mass was most common in this group, and a few lesions had internal spotty calcification. (2) The diffuse nodular type (21.59%, 19/88) has an extremely irregular shape and obscure boundary. In this study, the diameter of this type of lesion was 1–10 cm (17/88), and they were dominated by multiple hyperechoic nodules, which infiltrated the surrounding normal liver tissues along or around the intrahepatic portal vein/biliary tract branches and presented a “tree” or “map” distribution. These lesions had thickened and uneven internal echo, and fibrosis or calcification were common. (3) The mass type (21.59%, 19/88) has a spheroid shape, poorly defined boundary, and relatively large size. In this study, the diameter of this type of lesion was mostly >10 cm (14/88), with medium or low echo inside, peripheral “acoustic halo” and calcification visible, and obvious mass effect, with some of the lesions looking like metastatic tumors. (4) The fibrous calcification type has an irregular shape; poorly defined boundary; small size; mass-like high echo or strong echo followed by a sound shadow; coarse granular, pebble-like, annular, arc-like, cap-like calcification; and a large necrotic liquefaction with an obscure boundary, hyperechoic thick-walled structure in the periphery, extremely uneven thickness, rough inner wall, hypoechoic and weak echo in the center, and cave- or island-like changes.

No blood flow signals were detected in the above types of lesions, and truncated blood flow signals were seen around some of them. There were different degrees of calcification in all types of lesions. The detection rate of calcification in 80 cases was 48.75% (39/80).

All the 88 lesions involved into our study were classified into 5 groups based on imaging results. Lesions classified into localized hyper-, iso-echoic nodule, no well-defined, ovoid or irregular contour accounted for 18.1% (6/88), lesions classified into diffuse heterogeneous or homegeneous hyperechoic irregular contour, ragged edged accounted for 21.6% (19/88), lesions classified into homegeneous solid mass with large size, hypoechoic halo accounted for 21.6% (19/88), lesions classified into fibrocalcification, mixed heterogeneous echogenic pattern and irregular contour, including hailstorm pattern and small calcified lesions accounted for 30.7% (27/88), lesions classified into liquefaction necrosis accounted for 8% (7/88) (see Table 2).

We also classified the 88 lesions into 6 types according to “Kratzer Types” (22). 39 lesions were identified as hailstorm, 7 lesions were pseudocystic, 16 lesions were hemangioma-like, 19 lesions were metastasis-like, 4 lesions were ossification and the last 3 lesions were Undefined type (see Table 3).

**Table 3:** Sonographic appearances of 88 HAE lesions.

Sonographic Appearances	Number (%)
Localized hyper-, iso-echoic nodule, no well-defined, ovoid or irregular contour	16 (18.1)
Diffuse heterogeneous or homegeneous hyperechoic lesion, irregular contour, ragged edged	19 (21.6)
Homegeneous solid mass with large size, hypoechoic halo	19 (21.6)
Fibrocalcification, mixed heterogeneous echogenic pattern and irregular contour, including hailstorm pattern and small calcified lesions	27 (30.7)
Liquefaction necrosis	7 (8)
Kratzer Types	
Hailstorm	39 (44.3)
Pseudocystic	7 (8)
Hemangioma-like	16 (18.2)
Metastasis-like	19 (21.6)
Ossification	4 (4.5)
Undefined type	3 (3.4)

### PNM Status

The World Heath Organization’s Informal Working Group on Echinococcosis (WHO-IWGE) provide a “PCN” system for evaluating diagnosis performance and therapy outcomes (23). PNM-system denotes the extension of the primary mass in the liver (P), the involvement of neighbouring organs including lymph nodes (N), and metastases (M) (24). This system is similar to “TMM” system which was used to evaluate tumor and was widely used to distinguish stages of HEA before operation. PNM system could help patients understanding illness but didn’t play a key role in helping doctors to select the best treatment.

Expect for primary lesions, we found some lesions may correlate with HEA, including one mixed lesion spray, one solid mass at hilus lienis and one cavernous degeneration of portal vein. We didn’t found other cases that lesions metastasis to organs in abdominal cavity. Cases in our study didn’t received CT or MRI of lung and brain (see Table 4).

**Table 4:** PNM system for classification of human alveolar echinococcosis.

Classification		Number of patients (%)
P	Hepatic localisation of the parasite	
PX	Primary tumour cannot be assessed	–
P0	No detectable tumour in the liver	–
P1	Peripheral lesions without proximal vascular and/or biliar involvement	37 (46.25)
P2	Central lesions with proximal vascular and/or biliar involvement of one lobe^a^	28 (35)
P3	Central lesions with hilar vascular or biliar involvement of both lobes and/or with involvement of two hepatic veins	10 (12.5)
P4	Any liver lesion with extension along the vessels^b^ and the biliary tree	5 (6.25)
N	Extra-hepatic involvement of neighbouring organs or tissues	
NX	Not evaluable	–
N0	No regional involvement	78 (97.5)
N1	Regional involvement of contiguous organs or tissues	2 (2.5)
M	The absence or presence of distant metastasis	
MX	Not completely evaluated	78 (97.5)
M0	No metastasis^c^	–
M1	Metastasis	2 (2.5)

^*a*^ *For classification, the plane projecting between the bed of the gall bladder and the inferior vena cava divides the liver in two lobes.*

^*b*^ *Vessels mean inferior vena cava, portal vein and arteries.*

^*c*^ *Chest X-ray and cerebral CT negative.*

## Discussion

Because MR has unique advantages over other imaging examinations in the identification of non-calcified components inside the lesions, in a study on HAE cases with MR, Kodama et al. (25) found that these types of hyperechoic nodules were composed of multiple vesicle structures or small cysts of varying sizes (diameter: 1–20 mm), and the vesicles were the pathological basis of infiltrative growth of HAE lesions. They found that the early lesions were in a very active proliferative stage, and the edges of the vesicles constantly generated exospores to the periphery, forming an “inflammatory response zone” as a result of the immune response of the body (24, 26, 27).

The test on the HAE mouse model nine weeks after implantation infection also confirmed that the lesions detected via ultrasonography showed hyperechoic nodular changes (28). Under the pathological microscope, the center of the vesicle was liquefied necrotic tissue, and a large number of lymphocytes, macrophages, and cellulose-like substances gathered in the outer layer, forming an inflammatory response zone, which has a well-defined boundary with the normal liver tissue. According to the analysis, the hypoechoic lesions that should, in theory, have been visible via ultrasonography showed hyperechoic changes mainly because there was less liquid in the vesicles and the periphery was wrapped in thick cellulose-like substances, resulting in a strong reflection of ultrasonographic signals.

Contrast-enhanced ultrasound (CEUS), which could be helpful for speculating the activation of diseased tissues by evaluating blood perfusion on the level of microcirculation, have been widely used to support clinical medical as the application of new contrast agent (SonoVue).

Animal experiment confirmed that the sonographic infiltrative region in HAE lesion correlated with microvascular density (29).

A circular rim-like enhancement strip was visualized at the periphery during early arterial and delayed phases. The HAE strip was enhanced quickly and disappeared slowly. No contrast enhancement could be observed within the HAE lesions in the arterial, portal and delayed phases and the echo patterns were clearly lower than the normal liver tissue (the so-called “black hole” effect) (28, 30, 31).

CEUS could help doctors finding HAE lesions more efficiently and provides a more sensitive and accurate method for early diagnosis and identification of HEA especially lesions less than 2 cm (32), which are easily ignored via traditional US.

Recently, researchers studding CEUS pay more attention on “inflammatory response zone” of HEA but haven’t expounded the relationship between images and complex pathology of HEA.

Li S studied contrast parameters of different kinds of HAE and made a conclusion that peak intensity(PI), time to peak(TP) and mean transit time(MTT) of infiltrating, calculation differ to which of liquation type AE lesions, but there is no difference on area under the curve (33).

Ma SM showed that PI and AUC performed differently between central of lesion, inflammatory zones and surrounding tissues, but the results of experiments contradict with theory and this require more cases in further study (34).

Ehrhardt considered CEUS as a substitute for FDG-PET to estimate disease activity with the help of SonoVue, a new generation contrast agents, however, the lack of cases made the conclusion less convincing (35).

However, the development of HAE lesions in humans is a complex process. According to numerous studies, proliferation, necrosis, and calcification are the basic pathological changes of AE lesions. They can exist in the same lesion in different degrees at the same time. We also observed the coexistence of lesions in different stages of development on ultrasonograms of multiple liver lesions, which supported our inference. In addition, in terms of the diameter of the lesions alone, the diameter in the AE2 group (36.89 ± 20.12 mm) was less than in the AE1 group (55.48 ± 35.95 mm), which was less than in the AE3 group (80.00 ± 39.16 mm), which was consistent with this pathological development process. With the continuous increase in size and increased coagulation necrosis and liquefaction due to central ischemia, the lesions in the proliferative stage can also invade the bile duct or result in infection, producing complex ultrasonographic changes. The mixture of cystic and solid lesions forms island- or cave-like ultrasonographic changes, known as the pseudocyst sign by some scholars, and is characteristic of the AE3 type (32, 36). Theoretically, calcification is an external manifestation of calcium salt deposition in inactivated lesions and is another outcome of HAE lesions. According to Kratzer, the detection rate of calcification was 74.6%, which is much higher than our result (48.75%). This may be related to the fact that most of our samples were screening cases in the early and intermediate stages of the disease.

**Figure 1:**
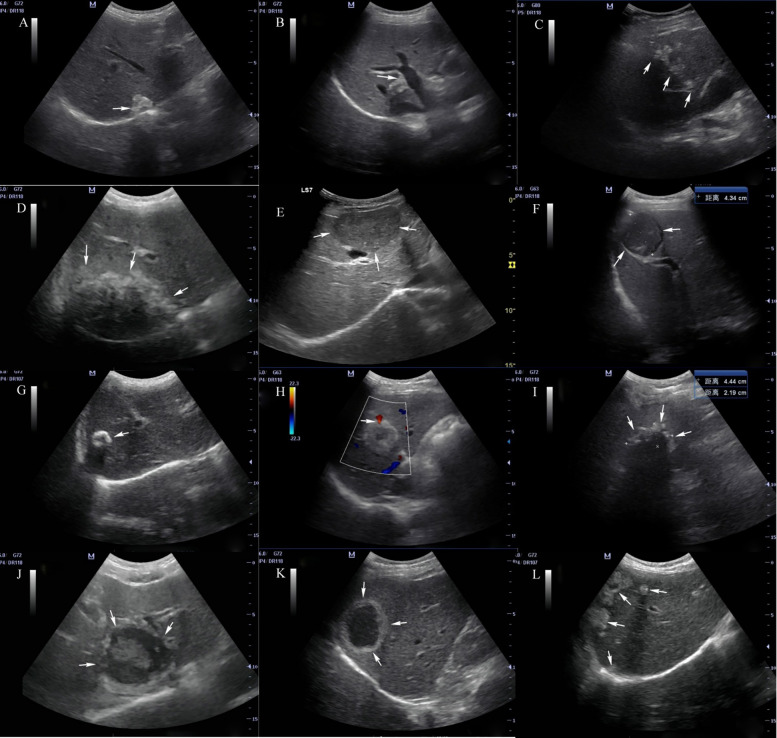
Ultrasonographic manifestations of hepatic alveolar echinococcosis Arrows in the figure: (**A**) hyperechoic nodular lesion; (**B**) angulation deformity of lesion edge; (**C**) treelike or beaded growth; (**D**) infiltration type; (**E**) mass type; (**F**) tumor-like sign; (**G**) calcification type; (**H**) vascular truncation sign; (**I**) “hailstorm” sign; (**J**) “island” sign; (**K**) “pseudocyst” type; (**L**) multiple AE lesions in liver.

At present, in addition to fine needle biopsy, we have no suitable examination method to monitor early HAE lesions with a diameter <3 cm (37). Therefore, it is of great significance to improve the understanding of sonographers on early HAE. Hemangioma-like changes and hailstorm sign are the early characteristics of HAE, and calcification is a common concomitant sign. Because the samples in this study came from clinically diagnosed cases and were not pathologically confirmed, further in-depth study with large samples is needed (see Figure 1).

## Data Availability

The original contributions presented in the study are included in the article/Supplementary Material, further inquiries can be directed to the corresponding author/s.
